# Characterization of the effects of defined, multidimensional culture conditions on conditionally reprogrammed primary human prostate cells

**DOI:** 10.18632/oncotarget.23363

**Published:** 2017-12-18

**Authors:** Lucas Tricoli, Aisha Naeem, Erika Parasido, John P. Mikhaiel, Muhammad Umer Choudhry, Deborah L. Berry, Iman A. Abdelgawad, Richard J. Lee, Adam S. Feldman, Chukwuemeka Ihemelandu, Maria Avantaggiati, Deepak Kumar, Stephen Byers, Rosa Gallagher, Julia Wulfkuhle, Emanuel Petricoin, Olga Rodriguez, Chris Albanese

**Affiliations:** ^1^ Department of Oncology, Lombardi Comprehensive Cancer Center, Georgetown University Medical Center, Washington, DC, USA; ^2^ National Cancer Institute of Egypt, Cairo, Egypt; ^3^ Massachusetts General Hospital Cancer Center, Boston, MA, USA; ^4^ Julius L. Chambers Biomedical/Biotechnology Research Institute, North Carolina Central University, Durham, NC, USA; ^5^ Center for Applied Proteomics and Molecular Medicine, George Mason University, Manassas, VA, USA; ^6^ Preclinical Imaging Research Laboratory, Georgetown University Medical Center, Washington, DC, USA

**Keywords:** prostate, cancer, primary tissue, reprogrammed cells, androgen

## Abstract

The inability to propagate human prostate epithelial cells indefinitely has historically presented a serious impediment to prostate cancer research. The conditionally reprogrammed cell (CRC) approach uses the combination of irradiated J2 mouse fibroblasts and a Rho kinase inhibitor such as Y27632 to support the continuous culture of cells derived from most epithelial tissues, including the prostate. Due to their rapid establishment and overall ease of use, CRCs are now widely used in a variety of basic and preclinical settings. In addition, CRCs were successfully used to clinically treat respiratory papillomatosis. Although both normal and tumor-derived prostate CRCs have been used to study the basic biology of prostate cancer and to test new therapies, certain limitations exist. We have previously reported that prostate CRCs form functional prostate glands when implanted under the mouse renal capsule. However in conventional culture, the prostate CRCs exist in an adult stem-like, transient amplifying state and consequently do not adequately recapitulate several important features of a differentiated prostate epithelium. To address these limitations, we previously described a transwell dish-based model that supported the culturing of prostate CRCs and the collection of cells and cell extracts for molecular and genetic analyses. Using normal and tumor-derived prostate CRCs, we describe the combined effects of the multi-dimensional transwell platform and defined culture media on prostate cellular proliferation, differentiation and signaling.

## INTRODUCTION

According to the American Cancer Society, the risk of prostate cancer (PCa) increases dramatically after age 50, and the usefulness and benefits of PSA testing for risk stratification has been vigorously debated [1]. As such, PCa remains the second leading cause of cancer related death in American men, and the American Cancer Society reports that approximately 180,000 new cases of prostate cancer and over 26,000 deaths occurred in 2016. Additionally, for patients whose disease has recurred after primary treatment, disease management remains complicated. Androgen deprivation therapy (ADT) is an effective first line therapy for locally advanced or metastatic disease. Unfortunately, once PCa recurs, the eventual development of castration-resistant prostate cancer (CRPC) remains an incurable disease and more effective therapies are needed [2].

Until recently, genetically engineered mice, patient-derived xenografts (PDX) and commercially available transformed cell lines have been the main models for prostate cancer research. While PDX mice are a useful model system, they can be difficult to establish, expensive to maintain and do not support the capacity for cellular modifications. In addition, PDX is a very low throughput approach, limiting its use in drug discovery and/or personalized PCa treatment. The widely used transformed PCa cell lines have yielded important information about prostate cancer progression and treatment. However, limitations exist with these cell lines in that they only represent a small component of the human disease [3] and, as a result, the critical need to develop more clinically relevant models persists.

Two recent advances in primary cell culture technologies, the organoid approach for prostate [4] and pancreatic [5] cancers and our conditionally reprogrammed cell (CRC) approach that effectively supports all epithelial tissues tested [6–8], have begun to address the need for sustainable cultures of primary human cells from both normal and cancerous epithelial tissues. Specifically, we and others have shown that the CRC approach, a combination of irradiated J2 mouse fibroblasts and a Rho kinase inhibitor (e.g. Y-27632), is an extremely robust methodology for enabling the indefinite propagation of primary cells from both normal and malignant epithelial tissues [7–15]. For example, we have used the CRC platform for the successful identification of the molecular basis of a rare form of cervical cancer [15] and importantly, for rapidly identifying an FDA-approved drug, vorinostat, to treat a case of recurrent respiratory papillomatosis in a patient who had failed a number of previous drug treatments [10].

CRCs have been widely used to study the basic biology of prostate cancer [16] and to define potential new therapies for its treatment [11–14]. In addition, CRCs have been generated and used for breast cancer research [17], for cystic fibrosis research [18, 19], to investigate drug sensitivity and resistance in lung cancer [20] attesting to the unprecedented broad applicability of this culture technology.

While this ability to derive primary cell cultures from prostate tissue has bona fide utility for prostate cancer research and therapeutic development, certain limitations currently exist. For example, our previous studies have found that both normal and tumor-derived CRC cultures contain cell populations that express basal cell markers, CD44 and Trop2 (CD49f), the luminal marker CD13 and a small population of CD117-expressing cells [16]. In addition, the expression of the basal cell marker cytokeratin 5 and the luminal cell markers cytokeratin 8/18 as well as low-level androgen receptor (AR) expression are all observed under conventional culture conditions [14, 16]. Perhaps not surprisingly, these cells also express the proliferative/basal cell marker p63 [16], indicating that prostate CRCs exist in a transient amplifying (TA), adult stem-like state with sub-optimal androgen sensitivity. Importantly, however, we have recently shown that long-term cultures of normal human prostate CRCs formed well-differentiated prostate glandular structures, and inclusive of nuclear localization of the AR and luminal prostatic secretions, when implanted under the mouse renal capsule [8]. These data clearly establish that conventional two-dimensional (2D) CRCs retain their lineage commitment and the ability to differentiate when placed in a permissive environment.

To support the growth of prostate CRCs in an *in vitro* environment that supports a more differentiated phenotype, we generated a filter-based multi-dimensional culture technique [21]. This transwell-dish culture method (TDCM) was initially described using cancer-derived CRCs from a patient with Gleason 6 prostate cancer [11]. When placed in the TDCM environment, subpopulations of cells were observed that expressed p63, suggestive of basal and proliferating cells, the AR, suggestive of luminal cells and in some cases both markers [21], perhaps indicative of transient cells. We also described the methods needed to recover CRCs from the filters and to collect nucleic acid and protein extracts from the cells in the TDCM system [21].

We have herein extended these initial studies to include both the normal and malignant (Gleason 6 and Gleason 8) prostate CRCs from two patients. We define the effects of TDCM on the expression of stem cell markers, and on proliferation and differentiation, and compare the TDCM cells to both CRCs grown in conventional two-dimensional cultures (i.e. plastic dishes) and to primary prostate tissue.

This study establishes that the TDCM platform enables the multi-dimensional culturing of normal and tumor-derived prostate CRCs and that these culture conditions supported the development of a more mature, prostate epithelial phenotype. Importantly, neither enhanced cell death nor senescence were observed after a week in the TDCM environment. Collectively, the combination of the patient-derived CRCs and the TDCM platform represents an innovative new tool for basic and translational studies of prostate cancer, as well as for investigations into normal prostate development.

## RESULTS

### Defined media selection

**Figure 1:**
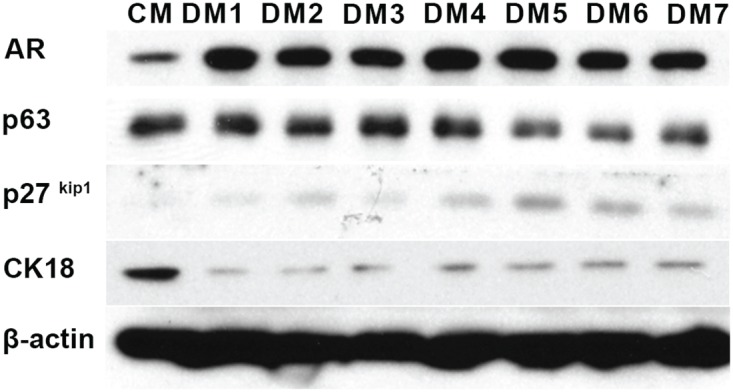
Defined media testing Western blotting of protein extracts of normal prostate CRCs from Patient 1. The cells were cultured for 7 days in either conditioned media (CM) or the defined medias (DM) listed in Table 1. CK18: cytokeratin 18. β-actin was used as a loading control.

In order to promote the transition of the CRCs from their transient amplifying, stem-like state to a more luminal phenotype, a series of defined media (DM) were formulated (Table 1). The normal prostate CRCs from Patient 1 were cultured for one week in conventional *in vitro* conditions, using either standard CRC conditioned media (CM) [22] or defined media (DM). Western blotting was performed to determine the expression of luminal markers AR and p27^kip1^ and the proliferative/basal marker, p63. β-actin was used as a loading control. As shown in Figure 1, DM5 and DM6, which contained Vitamin D3, known to regulate prostate cell proliferation [23], had the largest effects on p27^kip1^ and p63 expression. DM5 (referred to simply as DM) was chosen for all subsequent experiments.

**Table 1:** Composition of defined medias

DM1	30 nM citrate	1 nM zinc	5nM DHT	/	/	/	/
DM2	30 nM citrate	1 nM zinc	5nM DHT	20 ng/mL HGF	/	10 ng/mL TGFb	/
DM3	30 nM citrate	1 nM zinc	5nM DHT	20 ng/mL HGF	10 ng/mL IGF1	/	/
DM4	30 nM citrate	1 nM zinc	5nM DHT	/	10 ng/mL IGF1	10 ng/mL TGFb	/
DM5	30 nM citrate	1 nM zinc	5nM DHT	/	10 ng/mL IGF1	/	10 ng/mL VitD3
DM6	30 nM citrate	1 nM zinc	5nM DHT	20 ng/mL HGF	10 ng/mL IGF1	/	10 ng/mL VitD3
DM7	30 nM citrate	1 nM zinc	5nM DHT	20 ng/mL HGF	10 ng/mL IGF1	10 ng/mL TGFb	/

### The transwell dish culture method (TDCM)

**Figure 2:**
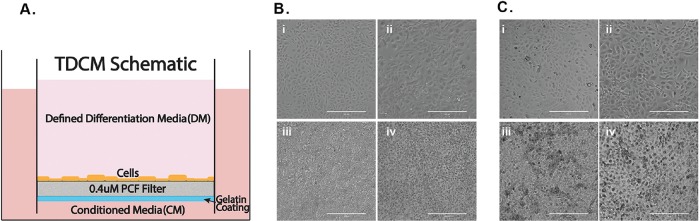
Culture conditions and cell morphology **(A)** Schematic of the TDCM (transwell dish culture method) platform. Micrographs of **(B)** normal and **(C)** tumor derived CRCs in i) conventional culture with CM, ii) conventional culture with DM, iii) TDCM with CM, iv) TDCM with DM. CM; conditioned media. DM; defined media. Scale bar, 400um.

As stated above, the TDCM system, shown schematically in Figure 2A, was initially defined using the Gleason 6 CRCs from Patient 1, wherein we found that the system supported both p63 positive basal-like cells and AR-positive luminal cells [21]. The methodological approaches required to recover the CRCs from the filters and to collect cell extracts from the inserts were also previously described [21]. Briefly, 6 × 10^5^ CRCs were seeded on the filter insert and CM containing 10 uM Y-27632 was placed in both the upper and lower chambers. After 7 days, DM or CM was applied to the top chamber and fresh CM was added to the bottom chamber. We have now extended our studies to include and compare both normal and tumor CRCs from Patients 1(Gleason 6) and 2 (Gleason 8) in order to more fully define the phenotypic effects of TDCM on both normal and tumor-derived prostate CRCs. The cytogenetic profiles of the tumor-derived CRCs are shown in Supplementary Figure 1.

### Phenotypic effects of CRCs growing under conventional and TDCM conditions

To define the morphological changes occurring in response to DM or growth in the TDCM system, images were collected following culture in the different conditions for seven days. As seen in Figure 2B and 2C, both the normal and tumor derived CRCs from Patient 2 show the typical, small cuboidal morphology of CRCs in conventional culture. Upon culturing in DM for one week, the cells became significantly larger (with an average cell diameter of 80.3 ± 25.2 um vs. 40.3 ± 9.8 um, p< 0.001) with a darker and more distinctive nucleus.

**Figure 3:**
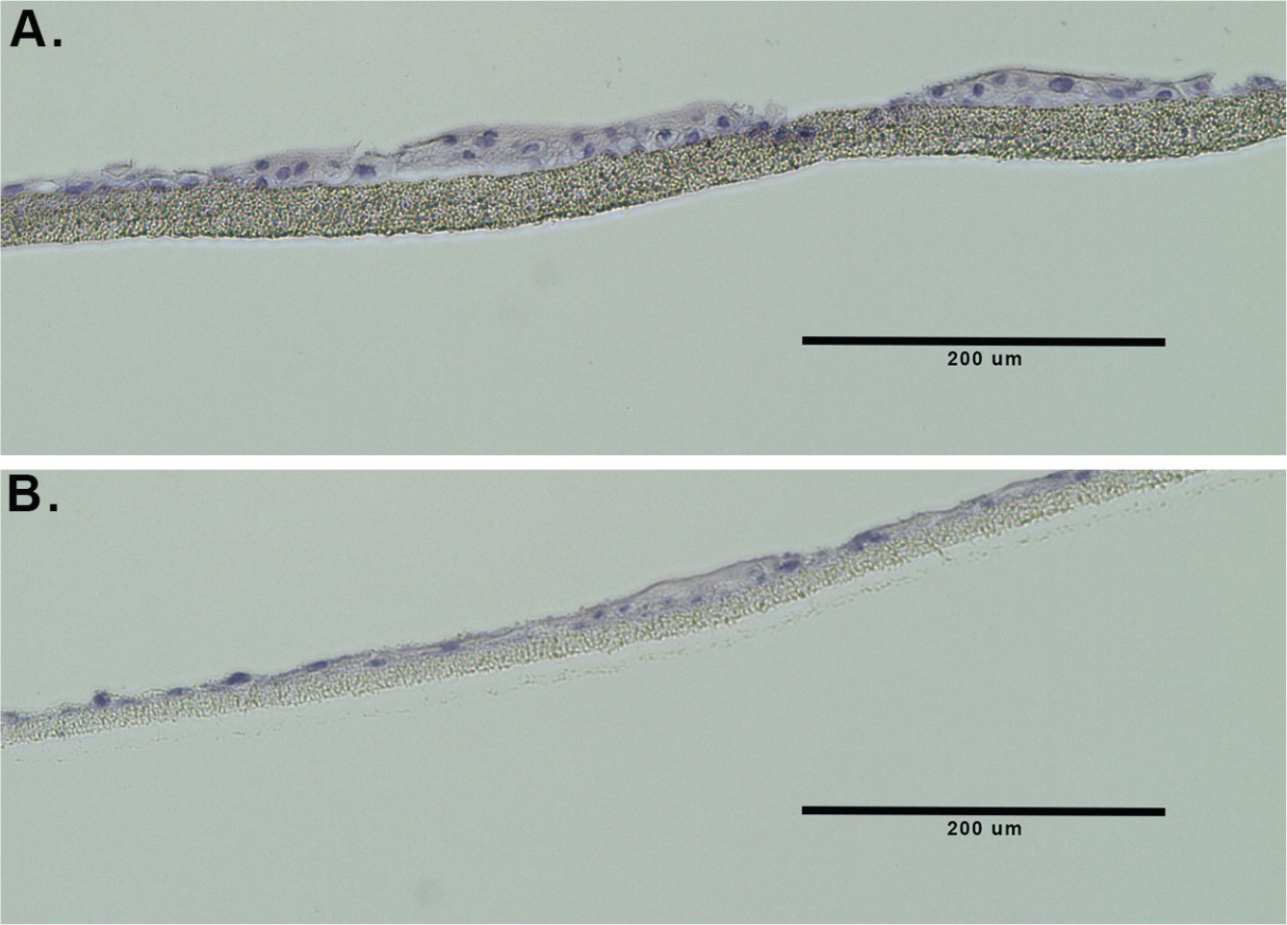
TDCM histology Hematoxylin and eosin- stained sections of formalin-fixed, paraffin-embedded CRCs from **(A)** normal prostate CRCs and **(B)** tumor-derived prostate CRCs.

The CRCs were next seeded into the TDCM system (as described in the Methods and in [21]). When cultured with CM in the bottom and top chambers, both the normal CRCs and tumor-derived CRCs appear tightly packed and aggregated on the filter surface (Figure 2B panels i and ii and Figure 2C panels i and ii, respectively). Similarly, when the CRCs were cultured with CM in the lower chamber and DM in the top chamber for seven days, they continued to display a tightly packed configuration across the filter surface (Figure 2B panels iii, iv and Figure 2C panels iii and iv). The filters were then carefully removed from the inserts as described in detail in [21] for histological analyses. Representative histological sections are shown in Figure 3. As previously seen with the cancer-derived CRCs from Patient 1, both the normal (Figure 3A) and malignant CRCs from Patient 2 (Figure 3B) formed stratified cultures that adhered to the filter surface, forming an organized layer of cells.

**Figure 4:**
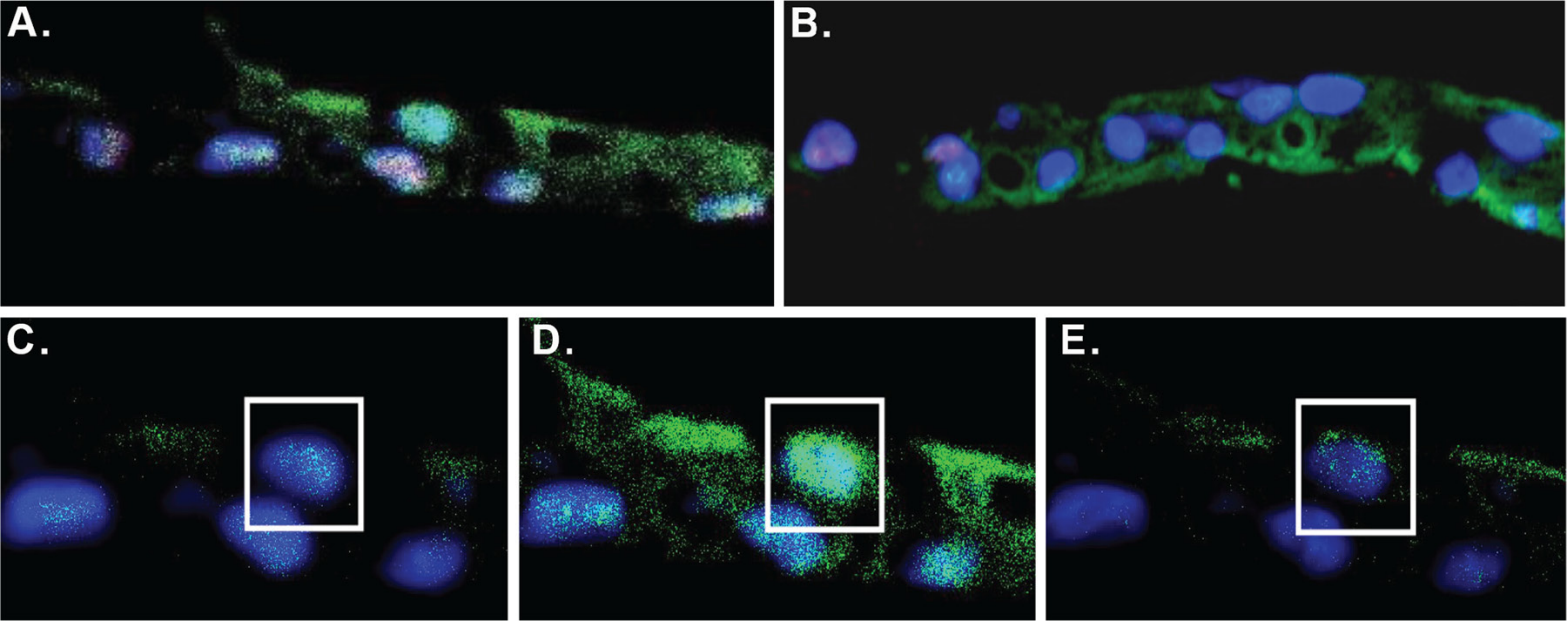
Immunofluorescent staining of CRCs **(A)** Normal and **(B)** tumor derived CRCs were cultured on the TDCM platform. Fixed paraffin sections were immunostained for the androgen receptor (green) and p63 (red). The cells were counterstained with DAPI (blue). **(C-E)** Confocal images of the sample in (A) taken at 1.5 micron increments.

Immunofluorescent (IF) staining of p63 and the AR was performed on both the normal and tumor-derived CRCs from Patient 2 (Figure 4). The cells were co-stained with DAPI to identify the nuclei. Similar to the tumor cells from Patient 1 [21], a subset of both the normal and tumor-derived CRCs from Patient 2 exhibited nuclear AR staining (green fluorescence), a feature consistent with prostate luminal cells. However since diffuse cytoplasmic AR staining was also observed, confocal imaging was performed and IF images captured in 1.5 um increments (Figure 4C-4E), confirming that a significant proportion of the AR signal was indeed localized within the nucleus.

In some cells, the IF staining also established the presence of nuclear p63 (red fluorescence), indicative of a proliferative, more basal-like layer of cells. A subset of the CRCs co-stained for both the AR and p63, which may be indicative of either transient amplifying cells or, in the case of the tumor-derived CRCs, proliferating tumor cells.

We conclude that the TDCM system supports the growth and stratification of normal and tumor CRCs with phenotypic features that resemble the prostate epithelium, including cells with luminal, basal and/or TA markers.

### Molecular analysis

**Figure 5:**
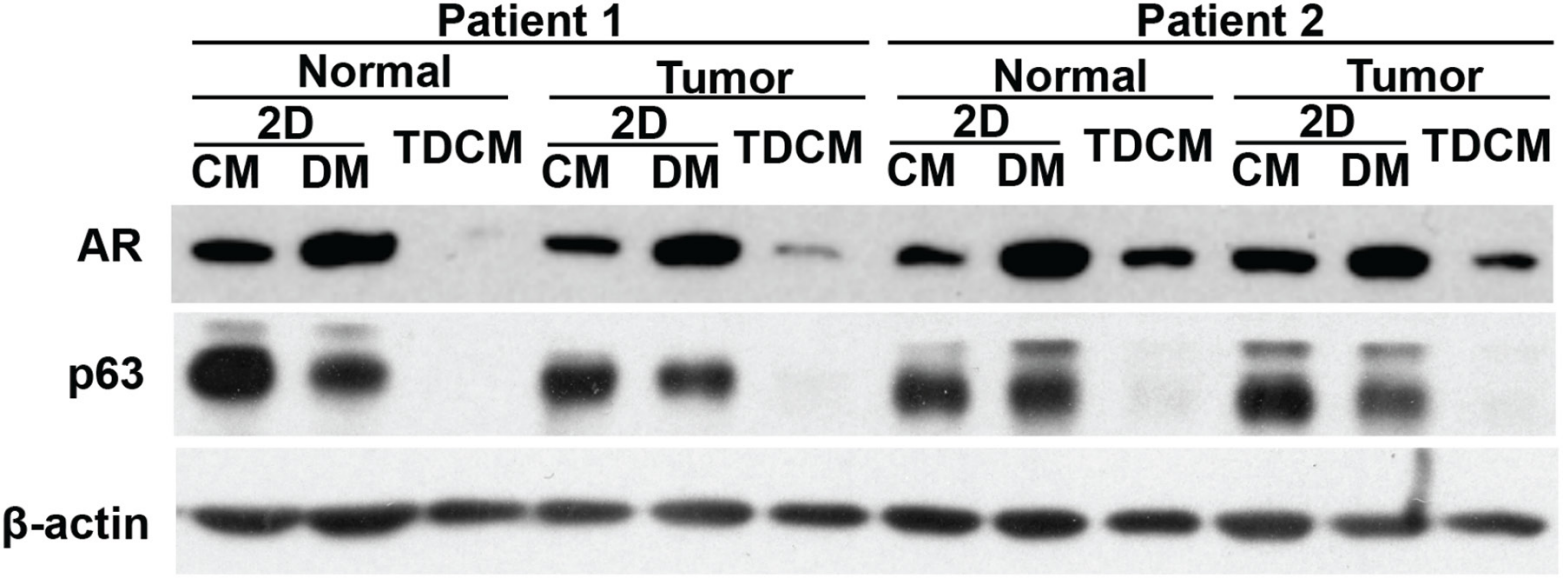
Conventional versus TDCM cultures Western blotting for the androgen receptor (AR) and p63. Normal and tumor CRCs from Patients 1 and 2 were cultured for 7 days as conventional cultures (2D) in either conditioned media (CM) or defined media (DM) or in the TDCM system with DM. β-actin was used as a loading control.

To define the effects of the TDCM on the protein markers indicative of proliferation and differentiation of the prostate CRCs, the levels of the AR and p63 were assessed by western blotting. As was seen in Figure 1, the levels of the AR increased and p63 decreased in the conventional cultures grown in DM (Figure 5). Interestingly, despite robust nuclear localization of the AR (Figure 4), total AR expression decreased in the TDCM/DM cells (Figure 5). The AR has been shown to possess an auto-feedback repressor function, acting via intron 2 of the *AR* gene [24] and this negative feedback loop may indeed be intact in the TDCM platform. A decrease in p63 expression was also observed.

**Figure 6:**
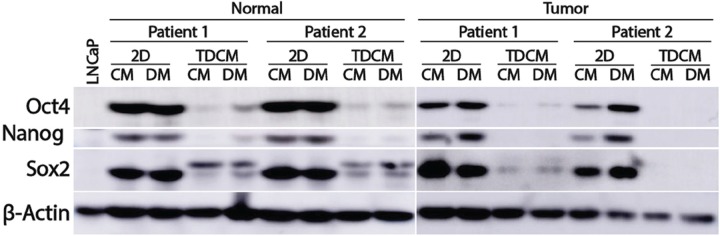
Culture conditions and effects on stem cell markers Normal and tumor-derived CRCs from Patients 1 and 2 were cultured for 7 days in either conventional cultures (2D) or in the TDCM system in either conditioned media (CM) or defined media (DM). β-actin was used as a loading control.

Human ectocervical CRCs express stem cell markers, such as Oct4, Sox2 and Nanog albeit at levels lower those found in mesenchymal stem cells [9]. To characterize expression of these markers in prostate CRCs, western blotting was performed. LNCaP cells were used as a negative control. When carried as conventional cultures in either CM or DM, the CRC lines all expressed Oct4, Nanog and Sox2 (Figure 6). Conversely, a significant loss of expression of these proteins was observed when cultured under the TDCM conditions, with the most pronounced changes observed in the tumor-derived CRCs (Figure 6). The TDCM conditions also resulted in an increase in PSA expression (Supplementary Figure 2). Collectively, the above data indicate that the TDCM/DM platform rapidly enables the CRCs to adopt a more differentiated phenotype with a concomitant suppression of the stem- and transient amplifying- like characteristics commonly associated with CRCs in conventional culture conditions.

### Comparison of CRCs to patient tissue samples

**Figure 7:**
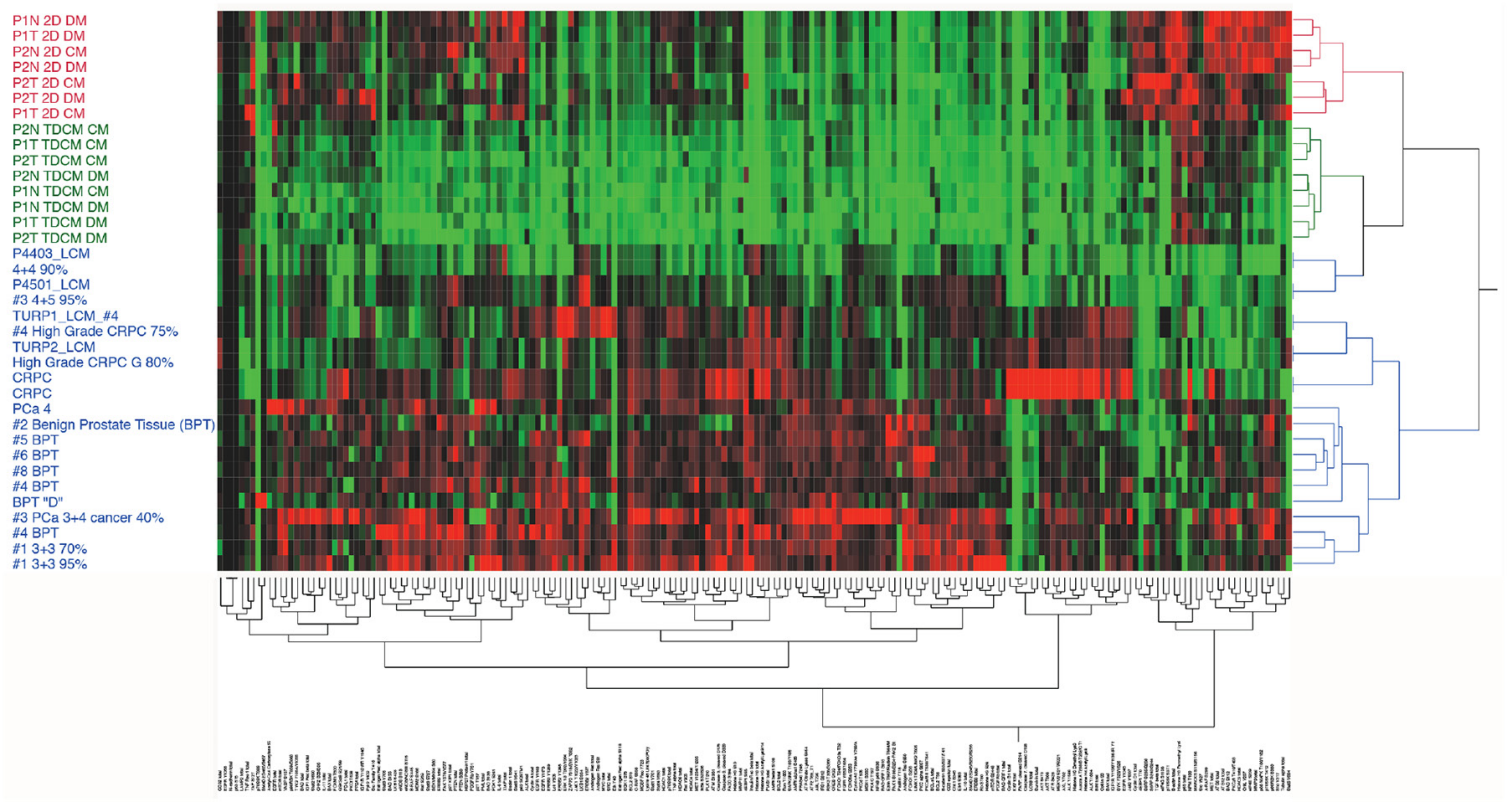
Unsupervised hierarchical clustering Reverse phase protein arrays were run on CRCs in either conventional culture or in the TDCM platform and compared to laser capture microdissected (LCM) primary prostate tissue. A panel of 159 protiens and phospho-proteins was used.

To better compare the CRCs to intact prostate tissue, reverse-phase protein array (RPPA) analyses were performed on extracts from the 2D and TDCM culture systems and compared against laser-capture microdissected (LCM) primary prostate cancer samples. The LCM and the RPPA pathway activation mapping analysis, covering 159 key signaling proteins and phosphoproteins from pathways known to be involved in tumorigenesis (growth, survival, apoptosis, autophagy, motility, inflammation, etc.) (Figure 7), were performed as previously described [25]. While the different sample types (LCM, TDCM, conventional CRCs) all clustered separately, the TDCM cells clustered more closely with the LCM samples. Furthermore, a number of key proteins, including cyclin D1 (which we have shown to repress AR activity [26]), receptor tyrosine kinases such as ALK and cMET as well as many members of the translation (e.g ribosomal S6 protein) and survival (e.g., BAD and LAMP2) signaling pathways were comparably expressed in the LCM and TDCM samples versus conventional CRC cultures (Supplementary Figure 3).

**Figure 8:**
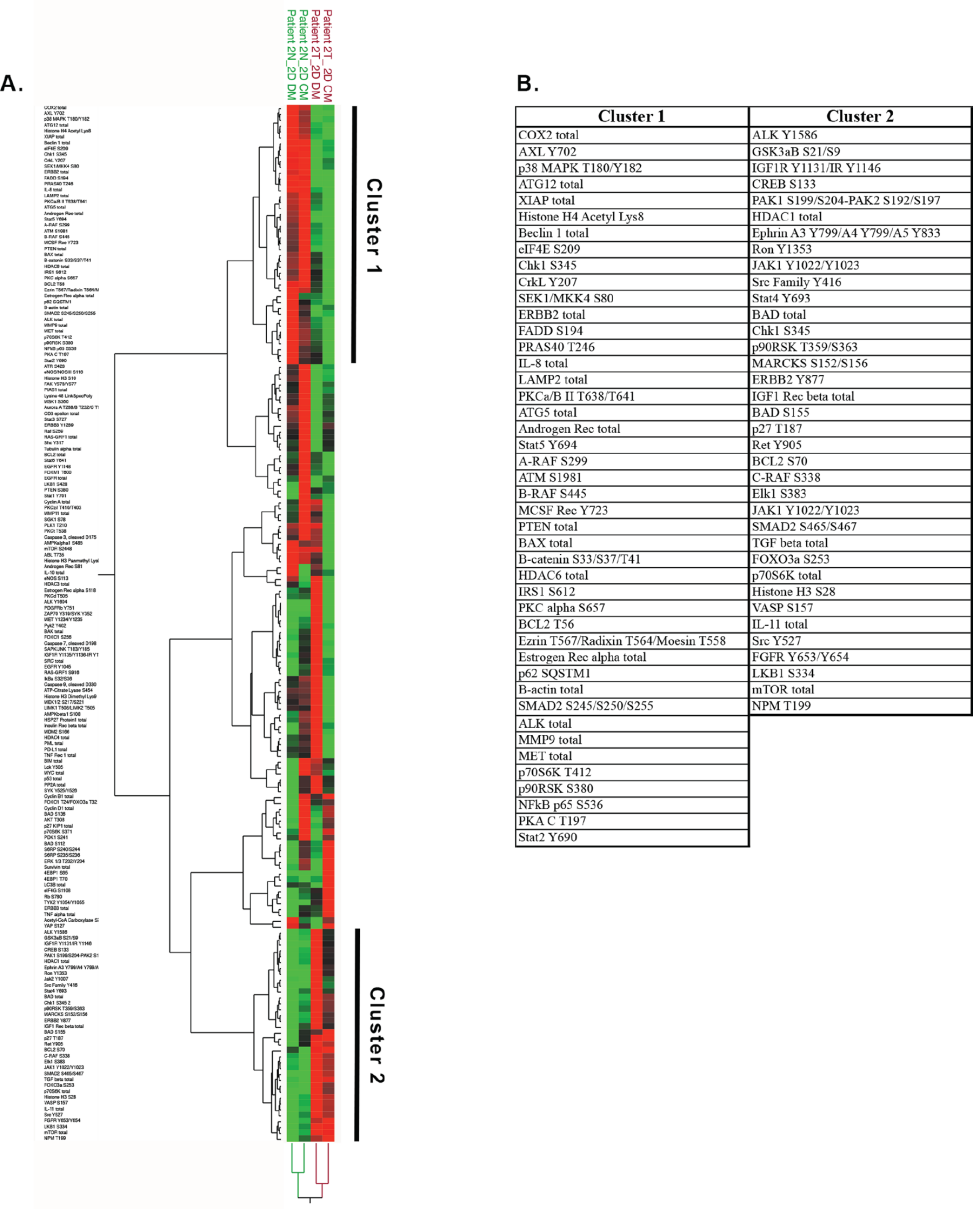
Protein analyses of cells in conventional culture **(A)** Unsupervised hierarchical clustering of the reverse phase protein array data from normal (N, green font) and tumor-derived (T, red font) CRCs from Patient 2, cultured in either CM (conditioned media) or DM (defined media). **(B)** List of proteins increased (Cluster 1) or decreased (Cluster 2) in tumor versus normal CRCs.

We next sought to define the cellular and molecular changes brought about by the culture media *per se*. A hierarchical two-way clustering analysis was performed on a subset of the data from conventionally cultured CRCs and focused on proteins involved in proliferation. As seen in Figure 8A, the cells from Patient 2 clustered based on tissue origin (normal vs tumor), with the proteins in Clusters 1 and 2 being a major component of the clustering that was independent of the culture media (Figure 8B). These data suggest that major components of the proteomic signature in the Gleason 8 CRCs are more strongly influenced by the genetic and molecular changes associated with prostate cancer than by the culture media.

**Figure 9:**
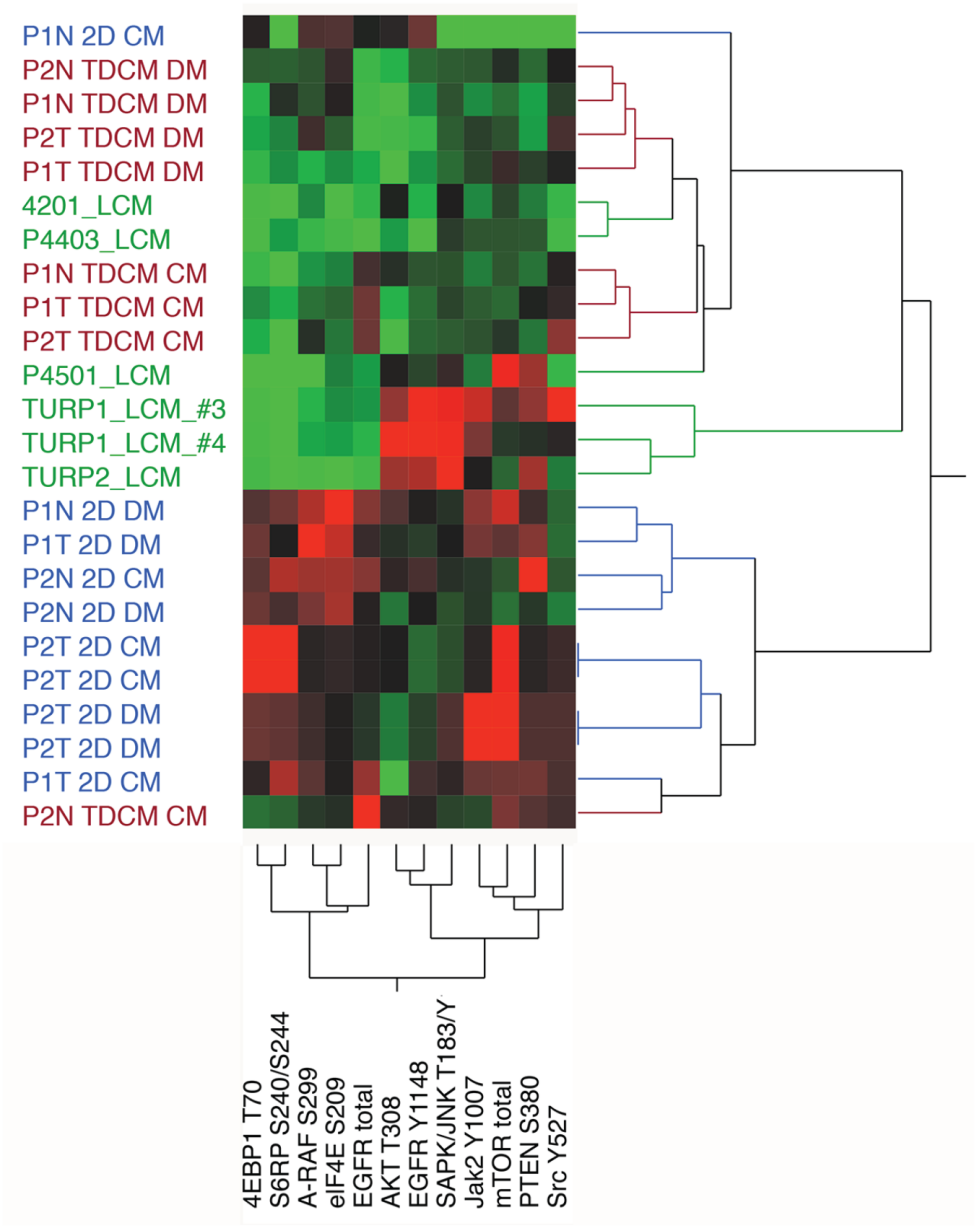
Unsupervised hierarchical analyses The protein array data from normal (N) and tumor (T) cells from Patient 1 (P1) and Patent 2 (P2,) were compared against LCM (laser capture microdissected) castrate resistant (TURP1, TURP2) and localized cancer samples. TDCM, conventional cultures (2D) and either conditioned media (CM) or defined media (DM) conditions were used.

Further unsupervised hierarchical analyses focused on proteins involved in MAPK and mTOR signaling and protein translation. When comparing the CRCs against localized PCa (samples 4201_LCM, P430_LCM and P4501_LCM) and CRPC (TURP1_LCM_#3, TURP1_LCM_#4 and TURP2_LCM), the TDCM cells clustered with the localized PCa samples while a subset of the proteins in the conventional cultures (e.g. total mTOR, Jak Y1007, PTEN S380) clustered with the TURP CRPC samples (Figure 9).

### Transcriptomic analyses

**Figure 10:**
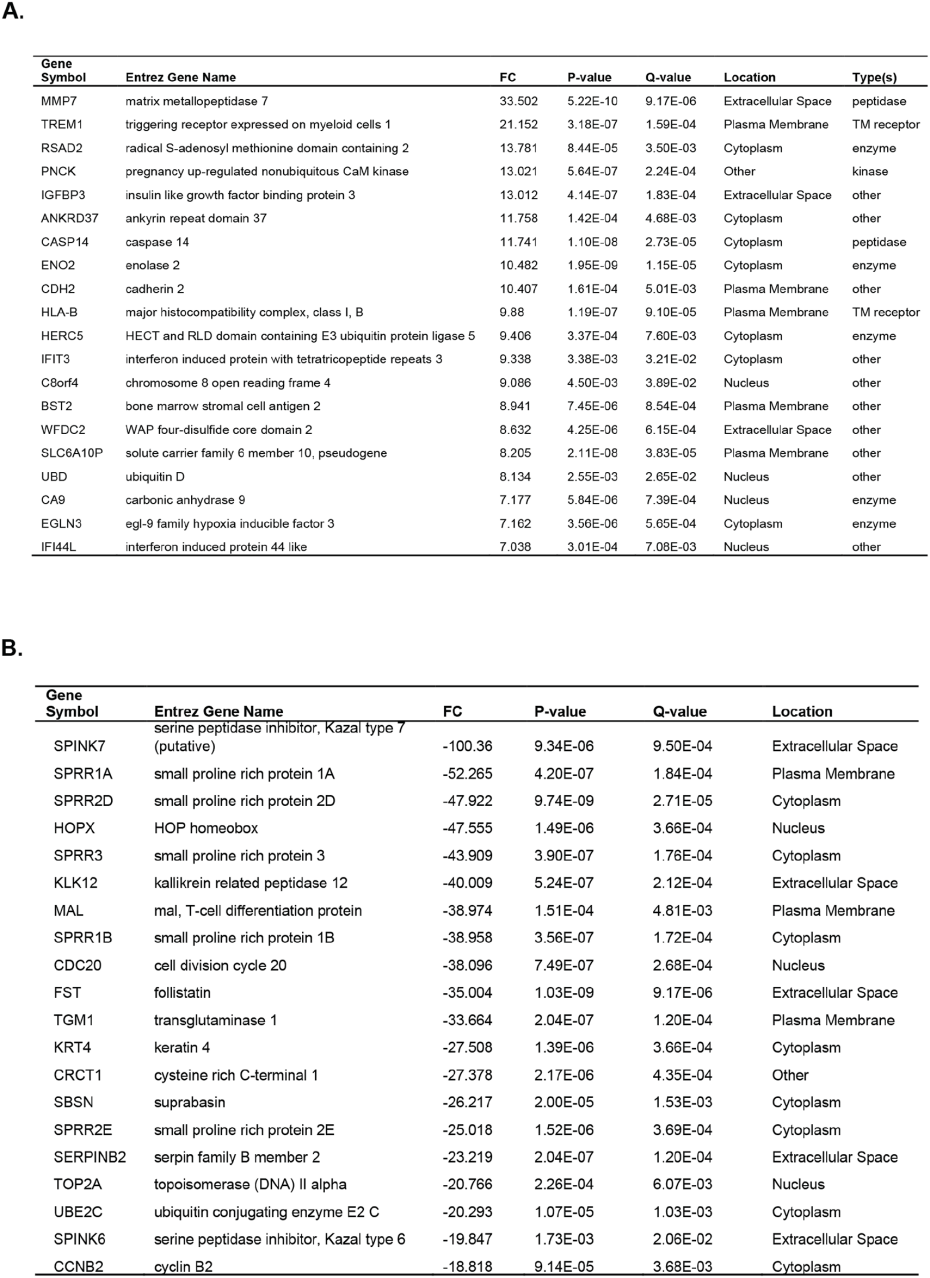
Microarray analyses performed on tumor cells from Patient 2 The top 20 genes **(A)** induced or **(B)** suppressed under TDCM vs conventional culture conditions are shown.

RNA microarray analyses were performed on Patient 2's tumor CRCs cultured under TDCM conditions vs. conventional CRCs and 157 genes were significantly upregulated (fold change > 1.5) and 3092 genes were significantly down regulated (fold change> -1.5, p-value <0.001, Q-value <0.015) in TDCM *vs*. conventional CRC culture conditions. The top 20 genes that were either induced or repressed in TDCM *vs*. 2D CM conditions are shown (Figure 10). Among the genes induced in the TDCM cells, two are associated with responses to hypoxia (*ANKRD37, EGLN3*), five are associated with immunity (*RSAD2, WFDC2, IFIT3, BST2, HLA-B*), and a vitamin D3 responsive gene (*TREM1*). In addition, there were multiple genes induced that are involved with cell and organelle structure and function (*PNCK, UBD, CDH2, HERC5*) and importantly, genes associated with the prostate, AR (*MMP7, CDH2, ENO2, IGFBP3*) and cancer (*IFIT3, IFI44L*). Among the genes repressed by TDCM are those involved in cell cycle progression (*CCNB2, CDC20, TOP2A, HOPX*) and in cell adhesion and migration (*SERPINB2, SPINK7, CAPN14*). In addition, stem markers and genes not normally associated with prostate were also suppressed (*MAL, KRT4, SPRR2E, SPRR2D. SPRR1A, SPRR1B, FST*). The induction of c-jun (Supplementary Table 1) was also observed under TDCM conditions.

**Figure 11:**
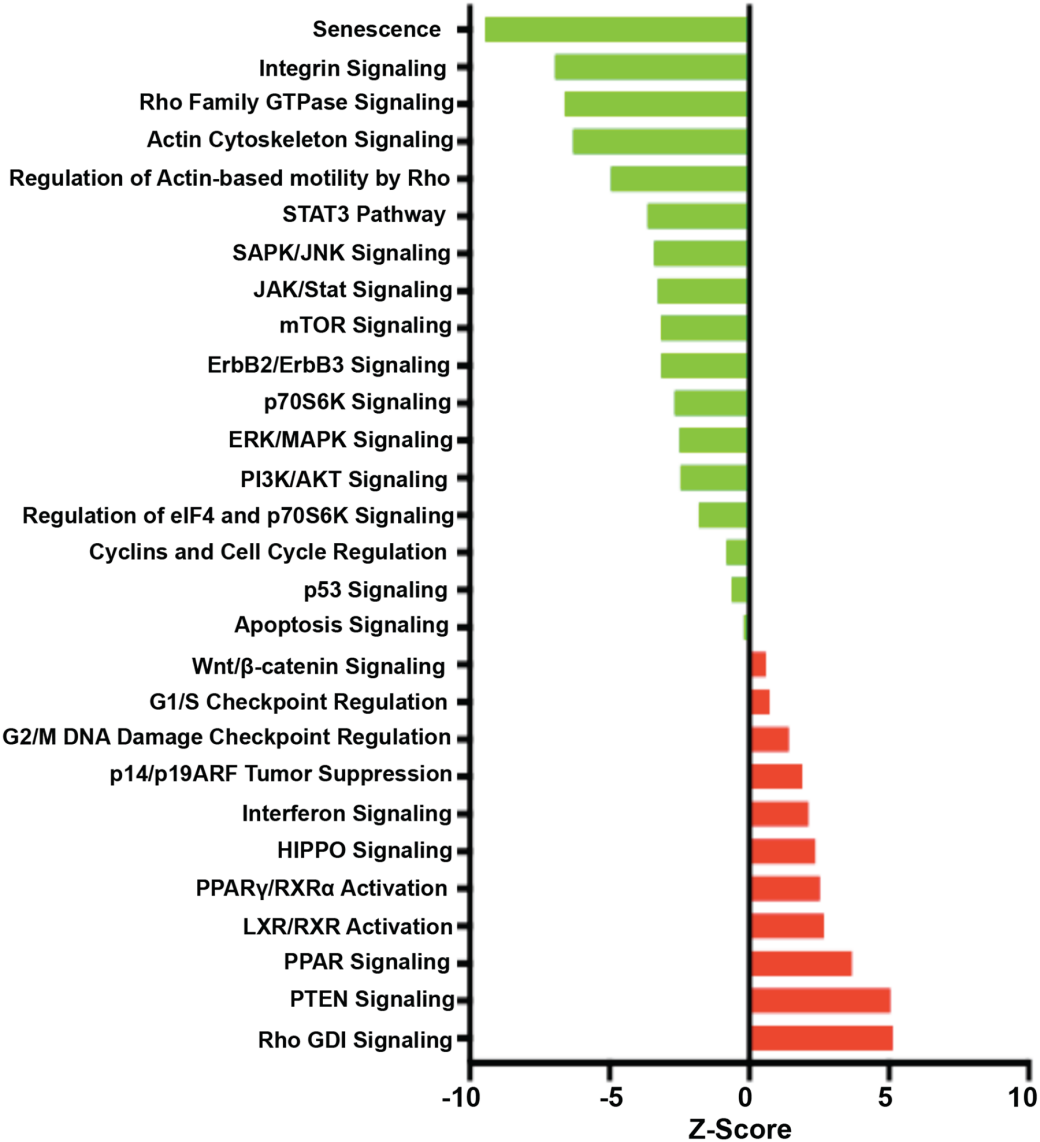
Signaling and regulatory pathway transcriptional regulation Impact analyses were performed on RNA from tumor-derived CRCs from Patient 2. The z-scores are shown as pathways induced (red) or repressed (green) in under TDCM conditions vs conventional culture conditions.

Finally, pathway analyses were performed using Ingenuity Pathway Analysis (IPA@Qiagen). Our IPA data established that the TDCM conditions significantly altered the levels of mRNAs that were enriched in canonical pathways such as senescence, Rac, PPAR, EIF2, HIPPO, p70S6K and mammalian target of Rapamycin (mTOR) signaling (Figure 11). It is important to note that the senescence-associated pathway was suppressed in our TDCM condition. Out of 455 genes associated with the senescence pathway, 185 genes were down regulated by TDCM *vs*. conventional culture versus 12 genes induced by TDCM, indicating that despite being in the TDCM system for over one week, the cells were healthy and viable. In addition, the activation of the HIPPO, G1/S checkpoint regulation, Rho-GDI, PPAR and β-catenin signaling were indicative of the overall improved health of the cells grown under TDCM conditions.

Thus our IPA analyses indicate that TDCM platform supports the continued vibrancy of the cells within the stratified cell layers.

## DISCUSSION

Existing models of continuous, long-term cultures of primary human prostate cells are currently restricted to either the R-spondin/organoids or our CRC-based methodology. While both of these approaches have unique strengths and weaknesses, perfecting these platforms through iterative modification and validation is vital to expanding their experimental and, ultimately, clinical use. We have therefore developed the TDCM system [21], and herein define its impact on the morphological and molecular phenotypes of normal and cancer-derived primary prostate cells.

The benefits of TDCM were apparent throughout the study. For example, while morphological changes, such as alterations in the typical cuboidal cell shape and increased size and darkening of nuclei, were detected within one week of conventional culture in DM, cells in the TDCM system adopted a stratified structure, resembling prostate epithelium. In addition, while the levels of the stem markers, Oct4, Nanog and Sox2, were not significantly altered by DM, both normal and tumor-derived CRCs cultured under TDCM conditions showed a significant to near complete loss in expression of these proteins, indicative of a shift away from transiently amplifying, stem-like cells. A decrease in overall AR expression was seen in TDCM cells, which may be attributed to the negative feedback loop that has been documented in the AR pathway [24]. Our analyses also predict an activation of nuclear AR under TDCM conditions, as the inhibition of cyclin D1 (Supplementary Figure 3) and the induction of c-jun (Supplementary Table 1) suggests that AR function is at least partially restored under these conditions, consistent with our immunofluorescent data (Figure 4) These data are further supported by changes in the androgen biosynthetic pathway, with increased expression of testosterone 17-beta dehydrogenase (Supplementary Table 1) and the observation that prostate specific antigen levels were increased in TDCM cells (Supplementary Figure 2). It is clear, however, that the terminal luminal differentiation achieved *in vivo* [8] remained partially incomplete.

While the TDCM proteome clusters predominantly with the primary tissue samples, there are key proteins that remain distinct. This raises the possibility that media conditions may need to be modified and/or that additional time in TDCM may be required. It also must be noted that the *in vivo* renal capsule transplantation experiments [8] were carried out over four or more months, and it is possible that the 7 days in DM were not sufficient for full engagement of the AR. The activation of Hippo signaling, PPAR signaling and wnt/β-catenin signaling indicated that cell's nutrient uptake capability and overall epithelial-associated gene expression profile was improved in TDCM conditions. Further, the near complete absence of a senescence-promoting profile, the reductions in apoptosis signaling and the lack of induction of a strong p53 signature, establish that the TDCM conditions provide a supportive environment for longer term experiments.

The normal prostate, as well as localized prostate tumors, develop and persist in a complex stromal microenvironment. For example, prostate ducts are surrounded by a fibro-muscular stroma, formed by urogenital sinus (UGS) and mesenchymal cells during embryonic development [27, 28]. The UGS and stroma are vital for differentiation of the ductal cells. The adult stroma, comprised of smooth muscle cells, fibroblasts and myofibroblasts, is critically important for the maintenance of normal prostate secretory function, and changes in the stromal architecture are associated with PCa [29]. A recent molecular study of the stroma of low and high Gleason grade PCa found that genes associated with osteoblasts, wound healing and metastasis, among others, were significantly induced in the “activated stroma” of high grade prostate cancers [30], supporting the hypothesis that the stroma supports the proliferation and possibly aggressiveness of PCa cells [31]. While co-culture of the CRCs with prostate stromal cells was not modeled in the current study, the TDCM platform is easily amenable to co-culture experiments. Future studies will assess the impact of stromal-epithelial interactions on the morphology and functionality of both normal and tumor-derived prostate CRCs.

## MATERIALS AND METHODS

### Cell lines and cell culture

Human radical prostatectomy samples were collected under the auspices and approval of the Georgetown University and Massachusetts General Hospital Institutional Review Boards. Following detailed pathological analyses that documented that the tissue sections collected were >70% tumor cells, the specimens were processed via protease dissociation as previously described [7]. Primary cultures were established at Georgetown University using the CRC method and maintained in co-culture or in conditioned media (CM) as previously described [22]. Genetic profiles, obtained by metaphase spreads on the normal and tumor-derived CRCs, were performed (Supplementary Figure 1). The Gleason 6 and 8 PCa lines remained diploid but showed increasing incidences of chromosomal aberrations, with the Gleason 8 cells from Patient 2 exhibiting numerous translocations and markers. Conversely the PCa3 CRC line, derived from a lymph node organoid line termed PCA3 in [32] was polyploid with extensive chromosomal gains and losses (Supplementary Figure 1). The normal prostate CRCs were diploid 46XY with no known translocations, deletions or cancer markers (not shown).

### Defined media

The defined media are comprised of the commercial prostate cell growth media (PrEGM, Cat. CC-3166) and additional supplements (Table 1). Defined media 5 (DM5), used throughout the study, contains 30 nM citrate, 1 nM zinc, 5 nM Dihydrotestosterone (DHT), 10 ng/mL Insulin-like Growth Factor 1 (IGF-1) and 10 nM vitamin D3 and is referred to throughout the study as DM.

### Transwell dish culture model (TDCM)

The CRC cultures were established and maintained as previously described by us [21]. Briefly, the prostate CRCs were grown on a 0.4 μm Millicell filter insert (Becton Dickinson) placed in a 6 well tissue culture dish. The filter inserts are first treated with a gelatin coating to help limit diffusion between the chambers [21]. Next, 6 × 10^5^ CRCs were seeded on the filter insert and CM containing 10 uM Y-27632 was placed in both the upper and lower chambers. After 7 days, DM or CM was applied to the top chamber and fresh CM was added to the bottom chamber. The cells were cultured for one week with frequent media changes. At the end of the study the inserts were removed for histology, immunostaining, protein, DNA and/or RNA extraction as described [21].

### Western blot analysis

Protein extracts were separated on 4-20% tris-glycine gels and electro-blotted onto PVDF membranes. Protein levels were assessed using antibodies against the AR (Santa Cruz Biotechnology, CA #816), p63 (Santa Cruz Biotechnology, CA #8431), p27 (Santa Cruz Biotechnology, CA #528), CK8/18 (Cell Signaling, Danvers, MA #4546), β-Actin (Cell Signaling, Danvers, MA #4967), Oct4 (Cell Signaling, Danvers, MA #2750), Nanog (Cell Signaling, Danvers, MA #4903) and SOX2 (Cell Signaling, Danvers, MA #3579).

### Immunohistochemistry and histology

The filters and cells were fixed in formalin overnight and processed by the Georgetown-Lombardi Histology and Tissue Shared Resource as previously described [21]. For immunohistochemical staining Five micron sections from formalin fixed paraffin embedded tissues were de-paraffinized with xylenes and rehydrated through a graded alcohol series. Heat induced epitope retrieval (HIER) was performed by immersing the tissue sections at 98°C for 20 minutes in 10 mM citrate buffer (pH 6.0) with 0.05% Tween. Slides were treated with 3% hydrogen peroxide, avidin/biotin blocking, and 10% normal goat serum and exposed to primary antibodies against the AR (1:80 dilution, Santa Cruz sc-816) overnight at 4°C, then against p63 (1:400 dilution, Santa Cruz sc-816) for 1 hour at room temperature. Slides were exposed to goat anti-mouse biotin-conjugated (Vector Labs) and goat anti-rabbit-488 (Thermofisher) secondary antibodies, and Cy3-SA (Thermofisher). Autofluorescence was quenched with Sudan Black and the slides mounted in Pro-Long Antifade with DAPI (Thermofisher). Consecutive sections with the primary antibody omitted were used as negative controls. Fluorescent images were captured using an QImaging camera on an Olympus BX61 microscope.

### RPPA proteome analysis

Cell pellets were isolated from the conventional or TDCM cultures and washed 3x with PBS as detailed by us [21]. Protein lysates were collected and Reverse Phase Protein Arrays (RPPA) were performed as previously described [25].

### Microarray analysis

RNA from conventional CRC cultures was isolated from cell pellets using an RNA easy kit (Qiagen Cat No. 74104). RNA was isolated from the TDCM cultures as detailed by us [21]. Expression analysis was performed in the Georgetown-Lombardi Genomics and Epigenomics Shared Resource using *HumanHT*-*12 v4* Expression BeadChip. Briefly, normalized data were imported into the R computing environment and analyzed using the Linear Models for Microarray Data package (LIMMA, 3.30.13 [33]) as part of the larger Bioconductor project (http://www.Bioconductor.org) [34]. A linear model was fit for the normalized log ratios of every gene using the ‘lmFit’ function within LIMMA to estimate all systematic variability in the data. Using functions in LIMMA, pairwise comparisons were performed between tumor cells cultured in TDCM differentiating media and conventional culture media to compute moderated t-statistics, log-odds ratios of differential expression (based on empirical Bayes for shrinkage of standard errors), and adjusted p-values using the Benjamini-Hochberg method [35].

### Functional analysis

Gene interaction networks, biological functions and pathway analyses were generated by Ingenuity Pathway Analysis (IPA) (Ingenuity Systems; Mountain View, CA, USA), with microarray data interpretation via grouping of differentially expressed genes into known functions, pathways, and networks primarily based on human and rodent studies. The identified genes were mapped to molecular functions and genetic networks available from the Ingenuity database. The significance was set at a *p*-value and adjusted *p*-value of 0.05 and a fold change of 1.5. From the complete data set of TDCM vs conventional CRC cells, 4367 molecules were eligible for core functional analysis by IPA.

The functional analysis identified the biological functions and the canonical signaling pathways that were most significant to the input data set. The significance of the association between the input data set and the functions or pathways was determined based on three parameters: (1) a ratio of the number of genes from the data set that map to the function/pathway divided by the total number of genes that map to the function/pathway (Overlap percentage) and (2) a *P*-value calculated using Fischer's exact test determining the probability that the association between the genes in the dataset and the function/pathway is explained by chance alone 3) z-score is a statistical measure of the match between expected relationship direction and observed gene expression. The calculated z-score predicts activation or inhibition of a pathway based on positive or negative z-score, respectively.

## SUPPLEMENTARY MATERIALS FIGURES AND TABLES





## Abbreviations

CRC: conditionally reprogrammed cells
PCa: prostate cancer
PSA: prostate specific antigen
AR: androgen receptor
TDCM: transwell dish culture method
DM: defined media
CM: conditioned media
2D: two dimensional
IPA: Ingenuity Pathways Analysis
LCM: laser capture microdissection
RPPA: reverse phase protein array
