# Supplementary material for: Characterization of the effects of defined, multidimensional culture conditions on conditionally reprogrammed primary human prostate cells
Source: Oncotarget. 2017 Dec 18;9(2):2193–207. doi: 10.18632/oncotarget.23363 (PMC5788632; doi:10.18632/oncotarget.23363)
Supplement: Supplementary file 1 [file oncotarget-09-2193-s001.pdf]

Characterization of the effects of defined, multidimensional culture conditions on conditionally reprogrammed primary human prostate cells

SUPPLEMENTARY MATERIALS

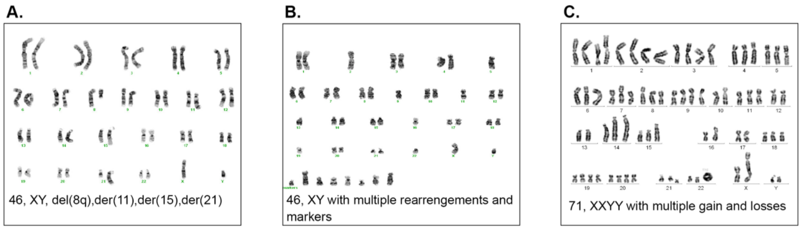

Supplementary Figure 1: Cytogenetic profiles. Metaphase spreads from conventional cultures of PCa CRCs. **(A)** Gleason's 6, **(B)** Gleason's 8 CRCs and **(C)** Castrate resistant Pca CRCs derived from PCa organoids (Gao, 2014).

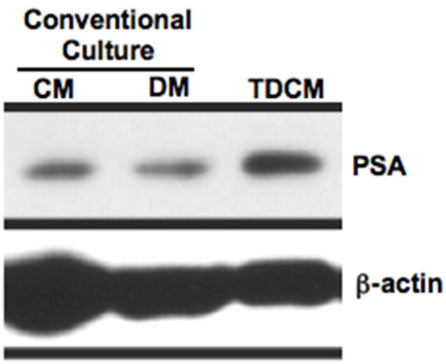

Supplementary Figure 2: Prostate Specific Antigen (PSA). PSA expression under conventional culture conditions vs TDCM. b-actin was used as a loading control. DM; defined media, CM; conditioned media.

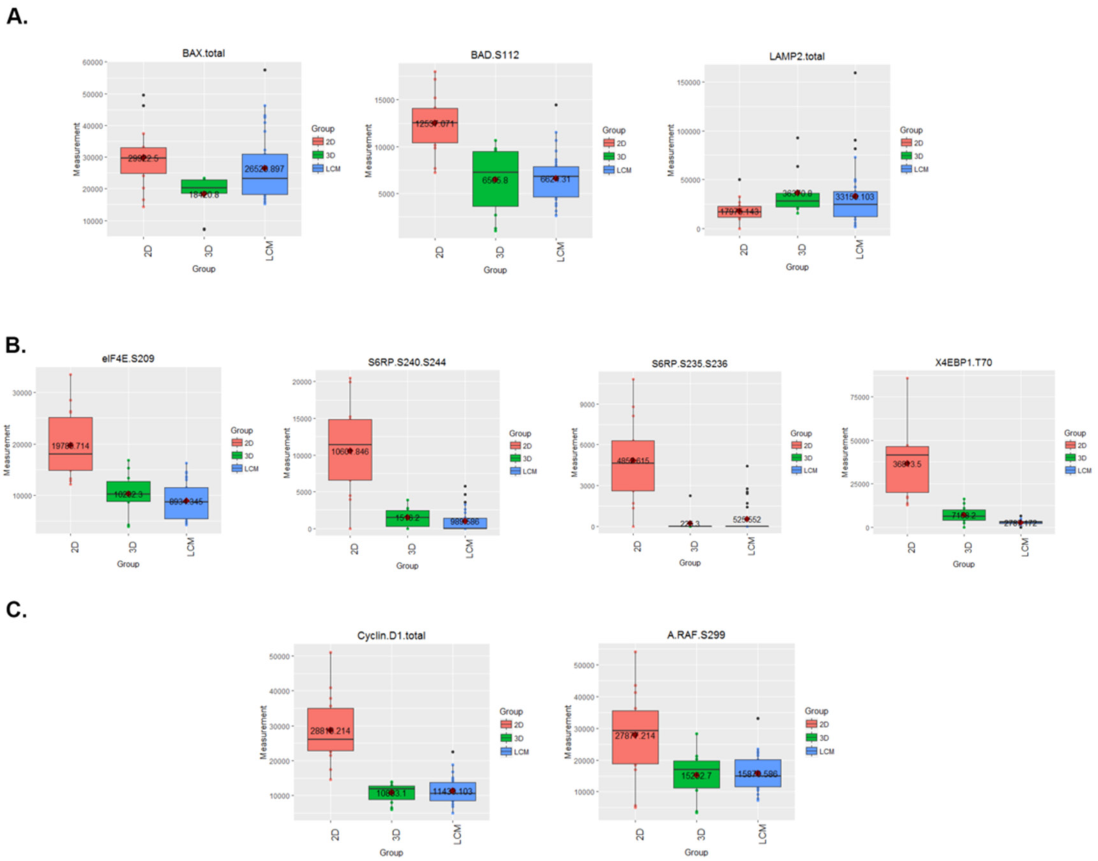

Supplementary Figure 3: Proteome profiles. Comparisons of protein array data on CRCs in conventional culture ((2D, red), TDCM (3D, green) versus laser capture microdissected prostate cancer samples (LCM, blue). Key proteins involved in **(A)** Apoptosis and autophagy, **(B)** Translation and **(C)** Cell cycle regulation are shown.

Supplementary Table 1: The list of differentially expressed genes Q<0.015 in Patient 2's tumor CRCs cultured under TDCM conditions vs. conventional CRC

See Supplementary File 1
